# Broccoli Byproduct Extracts Attenuate the Expression of UVB-Induced Proinflammatory Cytokines in HaCaT Keratinocytes

**DOI:** 10.3390/antiox13121479

**Published:** 2024-12-02

**Authors:** María Borja-Martínez, María A. Pedreño, Ana Belén Sabater-Jara

**Affiliations:** Department of Plant Biology, Faculty of Biology, University of Murcia, Campus de Espinardo, E-30100 Murcia, Spain; maria.borja@um.es (M.B.-M.); mpedreno@um.es (M.A.P.)

**Keywords:** broccoli byproducts, HaCaT keratinocytes, proinflammatory cytokines, reactive oxygen species, skin wound repair, supercritical fluid extraction, UVB radiation

## Abstract

Broccoli byproducts are an important source of bioactive compounds, which provide important benefits for human skin due mainly to their antioxidant and anti-inflammatory properties. The primary target of UVB radiation is the basal layer of cells in the epidermis, with keratinocytes being the most abundant cell population in this layer. Given the wide range of side effects caused by exposure to UVB radiation, reducing the amount of UV light that penetrates the skin and strengthening the protective mechanisms of the skin are interesting strategies for the prevention of skin disorders. This work aims to evaluate the protective mechanisms triggered by broccoli by-products extract (BBE) on HaCaT keratinocytes exposed to UVB radiation as well as the study of the regenerative effect of these extracts on the barrier of skin keratinocytes damaged by superficial wounds as a strategy to revalorize this agricultural waste. The results obtained revealed that the BBEs exhibited a high cytoprotective effect on the HaCaT exposed to UVB light, allowing it to effectively reduce the intracellular content of ROS, as well as effectively attenuating the increase in proinflammatory cytokines (*IL-1β*, *IL-6*, *IL-78*, *TNF-α*) and *COX-2* induced by this type of radiation. Furthermore, the BBE could be an excellent regenerative agent for skin wound repair, accelerating the migration capacity of keratinocytes thus contributing to the valorization of this byproduct as a valuable ingredient in cosmetic formulations.

## 1. Introduction

Human skin is the first barrier to external agents that cause damage to the body. In addition to UVB light, which is the main harmful factor facing this large organ, the skin can be exposed to other harmful light sources such as phototherapy systems that are mainly responsible for sunburn, photoaging, eye damage, generation of ROS and DNA damage, which can eventually cause skin cancer [1,2,3].

The primary target of UVB radiation is the basal layer of cells in the epidermis, with keratinocytes being the most abundant cell population in this layer, so they are considered the primary target of UVB light effects [4]. Keratinocytes act as skin immunocytes since they can secrete different cytokines, thus participating in the regulation of the immune response, so these cells have a major role in amplifying the inflammation cascade in skin diseases [5]. In addition, UVB radiation can increase gene expression and secretion levels of interleukins such as IL-1β, IL-6, the monocyte chemoattractant protein (MCP-1) and tumor necrosis factor alpha (TNF-α) among others [6].

Given the wide range of side effects caused by exposure to UV radiation, reducing the amount of UV light that penetrates the skin and strengthening the protective mechanisms of the skin are interesting strategies for the prevention of skin diseases.

To address this problem, the cosmetic industry is experiencing rapid growth worldwide due to the increasing awareness of beauty and a growing interest in health care, the protection of skin and cosmetic products. With the expansion of this sector, a new trend has emerged among consumers of natural, sustainable and “green” cosmetics, which has led to an increase in the world market based on plant extracts [7]. In this respect, plant byproducts from the agri-food industry are gaining attention and becoming more popular as a source of potentially functional cosmetic ingredients, since these agricultural wastes are an important source of value-added compounds, which provide important benefits for human health due mainly to their antioxidant properties [8,9,10,11]. Furthermore, from an economic and environmental point of view, the management of agro-industrial byproducts is being considered as a new target for the creation of novel strategies in the development of sustainable industrial processes since the valorization of these byproducts to produce high-value compounds is considered pivotal for sustainable growth based on the green concept of circular economy [12,13]. In this regard, several studies have demonstrated the protective effect of plant extracts against UVB light irradiation [14,15,16,17]. Thus, Mansuri et al. [18] showed that retinol (vitamin A) and palmitic acid present in broccoli leaves can serve as antioxidants to improve product performance against the aging effects of UV exposure or to enhance the aesthetic qualities of sunscreens. In addition, the content of flavonoids and phenolic compounds in these byproducts, which play a major role in sequestering free radicals, the main cause of numerous adverse effects in the skin, would make these extracts potential photoprotective agents. Furthermore, Shibata et al. [19] showed that pretreatment of a non-tumorigenic keratinocyte cell line (HaCaT) with sulforaphane (1–25 µM), an isothiocyanate found in Brassica vegetables, recovered the loss of viability induced by UVB light (50 mJ cm^−2^) and suppressed the secretion of IL-6, an inflammatory marker whose production increases after UVB light exposure. Previous studies from our group have also revealed that broccoli (*Brassica oleracea* L. var. italica, cv Naxos) byproducts (BBEs) obtained by supercritical fluid technology (SFE) are rich in bioactive compounds, with antioxidant properties which exert a protective effect against UVB radiation induced damage in HaCaT cells [8]. For this, our hypothesis is that BBEs are capable of protecting epidermal keratinocytes from UVB radiation through the activation of the signaling pathways for inflammatory processes mediated by ROS, which is considered to be the major cause of photoaging after the acute exposure of the skin to UVB radiation. Thus, to gain more knowledge about the protective mechanisms triggered by BBEs in human keratinocyte cells exposed to UV light, the present study aimed to evaluate some of the molecular mechanisms involved in the antioxidant and anti-inflammatory effects of BBEs on UVB-irradiated HaCat cells, as well as the study of the regenerative effect of these extracts on the barrier of skin keratinocytes damaged by superficial wounds as a strategy to revalorize this agricultural waste.

## 2. Materials and Methods

### 2.1. Preparation of Extract

Broccoli (*Brassica oleracea* L. var. italica, cv Naxos) byproducts extract (BBE) was obtained by SFE as it was previously reported [8]. Briefly, dried ground broccoli byproducts (15 g) were placed in the pressurized extraction vessel in a Waters Prep Supercritical Fluid Extraction system (SFE-100Waters^®^, TharSFC, Thar Technologies, Inc., Pittsburgh, PA, USA). The equipment was coupled with P-50 pumps (CO_2_ and ethanol as co-solvent), two pressure heating exchangers (low and high), two pressurized collection vessels, and three automated back-pressure regulators. The SFE system was connected to an Accel 500 LC chiller by Thermo Scientific (TharSFC, Thar Technologies, Inc., Pittsburgh, PA, USA). SFE was performed at 40 °C and 443 bars in a dynamic mode with 7% ethanol at a total flow rate of 31 g min^−1^ during 68 min. The resulting extract was evaporated at 35 °C in a rotavapor (Rotavapor^®^100, BÜCHI Labortechnik AG, Flawil, Switzerland) and stored at −20 °C until analyses. For the bioactivity assays, the dried BBEs were reconstituted in dimethylsulfoxide at 20 mg of extract mL^−1^, filtered through 0.2 µm nylon syringe filters and stored at −20 °C.

### 2.2. Characterization of Bioactive Compounds in BBE

The identification and quantification of bioactive compounds in the BBEs were performed following the methodology previously reported by our research group [8] using high-performance liquid chromatography (HPLC-DAD) and gas chromatography (GC), equipped with a mass selective detector (Agilent Technologies 5973) (GC-MS). Prior to the analysis, the dried extracts were reconstituted in ethanol (1000 µg BBE mL^−1^) and filtered through 0.2 µm nylon syringe filters. The identification and quantification of carotenoids and chlorophylls were carried out by HPLC on a Jasco LC-NetII/ADC system (Jasco, Easton, MD, USA) equipped with a photodiode array detector, an autosampler, a control module and a Zorbax Eclipse XDB-C18 reverse-phase column (250 × 4.6 mm i.d., 5 µm particle size; Agilent Technologies, Palo Alto, CA, USA). Acetonitrile/water (9:1; *v*/*v*) and ethyl acetate were used as phases A and B in a gradient elution over 35 min. The injection volume was 20 µL, and the flow rate was 1 mL min^−1^. GC-MS analysis, for the identification and quantification of phytosterols and α-tocopherol, was performed on an Agilent Technologies 6890 Network GS System, equipped with a mass selective detector (Agilent Technologies 5973). For GC-MS analysis, an Agilent 19091 S-433HP-5MS capillary column (30 m × 0.25 mm). Helium, at a constant flow rate of 0.1 mL min^−1^, as carrier gas was used. Bioactive compounds were identified by comparison with commercially available standards. The chromatograms obtained from HPLC-DAD and GC-MS and the most abundantly identified and quantified compounds in the BBEs are presented in Figures S1 and S2 (Supplementary Materials), as detailed in the publication [8]. Total phenolic content was determined spectrophotometrically using the Folin–Ciocalteu method [20] on a UV-VIS spectrophotometer (JASCO V-730, Easton, MD, USA) using a calibration curve of gallic acid standard, as previously described by Borja-Martínez et al. [8]. The total bioactive compound content in the BBE was 237.38 ± 17.42 mg g^−1^ BBE, expressed as the sum of β-carotene (62.24%), chlorophylls (17.22%), total phenolic compounds (12.31%), phytosterols (5.34%) and α-tocopherol (2.39%).

### 2.3. Antioxidant Capacity of the BBEs

The antioxidant capacity of the BBE was determined using the Trolox Equivalent Antioxidant Capacity (TEAC) assay according to the protocol described by Borja-Martínez et al. [8]. The reaction medium contained 2 mM ABTS*, 45 μM H_2_O_2_, 330 ng mL^−1^ horseradish peroxidase (HRP VI) and 12 mM sodium acetate buffer at pH 5.0. The activity of BBEs on ABTS* was measured spectrophotometrically at 414 nm using a Jasco V-730 spectrophotometer (Easton, MD, USA). The total antioxidant capacity of the BBEs was found to be 338.69 ± 31.95 mg Trolox g^−1^ BBEs.

### 2.4. HaCaT Cell Culture Maintenance

HaCaT cells (CLS Cell Lines Service (Eppelheim, Baden-Württemberg, Germany)), a human immortalized non-tumorigenic keratinocyte cell line, were maintained in Dulbecco’s modified Eagle’s medium (DMEM) supplemented with 4.5 g L^−1^ glucose, 2 mM L-glutamine and 10% fetal bovine serum (FBS) at 37 °C in a humidified incubator containing 5% CO_2_. For this, HaCaT keratinocytes were seeded in 96-well microplates at a density of 5 × 10^3^ cells per well and incubated until the cells reached 70–80% confluency.

### 2.5. Cell Viability Assay

The viability of HaCaT cells pretreated with BBE (ranging from 0 to 50 µg mL^−1^) and irradiated with UVB was determined using the MTT [3-(4,5-dimethylthiazol-2-yl)-2,5-diphenyltetrazoliumbromide] assay as previously described by Borja-Martínez et al. [8]. For this, the medium was completely removed from the wells and replaced with 200 µL of MTT (1 mg mL^−1^ in serum-free DMEM) and incubated for 4 h at 37 °C and 5% CO_2_. The results were expressed as a percentage of viable cells compared to the non-treated cells.

### 2.6. Determination of Intracellular ROS Production

To evaluate the intracellular ROS levels, the fluorescent oxidizing radical species-sensitive dye 2′,7′dichlorodihydrofluorescein diacetate (DCFH-DA) (Sigma-Aldrich, Hamburg, Germany) was used. HaCaT cells (5 × 10^3^ cells per well) were grown for 24 h in fluorescence microtiter 96-well plates in DMEM complete medium at 37 °C and 5% CO_2._ After cellular attachment, cells were pretreated with different concentrations of BBE ranging from 0.1 to 50 µg mL^−1^ in complete DMEM medium for 24 h. Then, the medium containing the BBE was discarded, and cells were incubated for 30 min with 10 μM DCFH-DA in phosphate buffer solution (PBS) at room temperature in the dark. Subsequently, cells were irradiated with 50 mJ cm^−2^ UVB light (Vilber Louvert Biolink—BLX UV Crosslinker, Eber-Hardzell, Germany) as previously described by Borja-Martínez et al. [8]. After UVB irradiation, the probe was washed twice with PBS and replaced with complete DMEM medium. Immediately after that, fluorescence intensity was determined using a microplate reader Omega FluoSTAR (BMG Labtech, Ortenberg, Germany). Relative ROS production was determinate by the oxidation of DCFH to 2′,7′-dichlorofluorescein (DCF) using an excitation wavelength of 485 nm and emission wavelength of 520 nm. Changes in DCF fluorescence intensity were expressed as a percentage compared with control cells (non-treated and non-irradiated cells).

### 2.7. RNA Isolation and Quantitative Real-Time RT-PCR

Total RNA was isolated from HaCaT cells which were previously seeded into 6-well plates at a density of 1.5 × 10^5^ cells per well in complete DMEM medium. After 48 h of incubation to allow for attachment, the cells were pretreated with different concentrations of BBEs ranging from 0.1 to 50 μg mL^−1^ in complete DMEM medium for 24 h. Then, keratinocytes were exposed to 50 mJ cm^−2^ UVB, as described above. After 2, 8, 14 and 24 h of UVB irradiation, the culture medium was removed and the cells were lysed to obtain RNA using the RNEasy MiniKit extraction kit (Qiagen, Hilden, Germany), following the manufacturer’s specifications. Once the cells were lysed and the RNA was extracted, the samples were frozen in liquid N_2_ and stored at −80 °C until use. In addition, to eliminate possible remains of genomic DNA, the samples were subjected to DNase treatment, using the RNase-Free DNase Set kit (Qiagen, Hilden, Germany) following the manufacturer’s recommendations. The yield and purity of the RNA was determined using a NanoDrop ND-2000 spectrophotometer (Thermo Fischer Scientific, Waltham, MA, USA). Starting from 1 µg of purified RNA, the cDNA was synthetized using the RevertAid First Strand cDNA Synthesis Kit (Thermo Fischer Scientific, Waltham, MA, USA) according to the manufacturer’s protocol.

Real-time PCR analysis was performed in an Applied Biosystems QuantStudioTM 5 Flex Real-Time PCR system (Thermo Fisher Scientific, Waltham, MA, USA) in 10 μL, using the 2X Power SYBR Green PCR Master Mix (Applied Biosystems, Carlsbad, CA, USA). It was used with 5 μL SYBR Green reaction mix, 51 nM forward and reverse gene-specific primers (Table 1) and 33 ng of cDNA template. PCR cycle conditions were 2 min at 50 °C, 10 min at 95 °C, followed by 40 cycles of 15 s at 95 °C and 1 min at 60 °C. For each gene, expression values were normalized to the *GAPDH* as reference gene. All experiments were performed in triplicate.

### 2.8. Scratch Wound Healing Assay

HaCaT cells were seeded into 24-well microplates at 5 × 10^4^ cells per well and maintained in complete DMEM medium until reaching ~90% confluency. Then, the BBEs were added reaching a final concentration of 0.1, 1, 10 and 50 µg mL^−1^ in the wells and, after 24 h, a wound was made in each well using a sterile micropipette tip. Quercetin (30 µg mL^−1^) was used as the positive control. Immediately, the medium was discarded, and cells were washed with PBS to remove any detached cells. Cells were then supplemented with complete medium with or without the extract at the same concentrations as above described. Pictures were taken at 0, 6, 24 and 48 h after the wound was made using a bright-field inverted microscope Zeiss Axio Observer 7 (Zeiss, Oberkochen, Germany). Treatments were made by duplicate and the migration was evaluated in two different areas of each well tracing the wound area using ImageJ software, version 1.51j8 (Bethesda, MD, USA). The percentage of wound closure and the migration speed were calculated using the following Equations (1) and (2):

(1) $$\%\;Wound\;closure=\frac{Wound\;area\;\left(T0\right)-Wound\;area\;(Tx)}{Wound\;area\;(T0)}\times 100$$

(2) $$Migration\;speed\;\left({mm}^{2}/h\right)=\frac{Wound\;area\;\left(Tx\right)-Wound\;area\;(T0)}{Tx}$$

### 2.9. Statistical Analysis

Data were analyzed by two-way analysis of variance (ANOVA) followed by Tukey’s honest significance test to examine the significance of the observed differences using the SPSS package, version 22 (SPSS Inc., Chicago, IL, USA) version 22, and *p*-values <0.05 were considered as statistically significant.

## 3. Results

### 3.1. Effect of BBE on Intracellular ROS Production in UVB-Exposed HaCaT Keratinocytes

**Figure 1:**
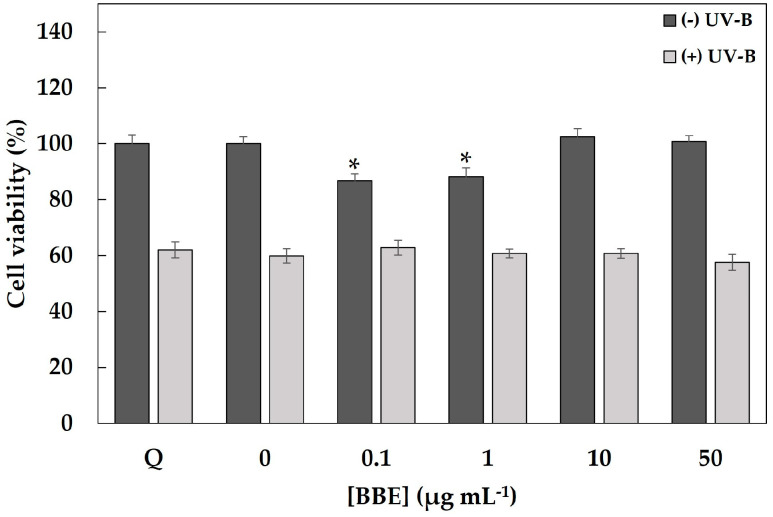
Effect of different concentrations of broccoli by-product extracts (BBE; 0, 0.1, 1, 10 and 50 µg extract mL^−1^) on the viability of UVB-irradiated (50 mJ cm^−2^) HaCaT cells. Cell viability was determined after 24 h of UVB-irradiation by the MTT method and expressed as percentages of the control. Values are given as the mean ± SD of six replicates. Asterisks denote significant differences according to the Tukey test (*p* < 0.05) in non-irradiated cells. Q: Quercetin (30 µg mL^−1^).

**Figure 2:**
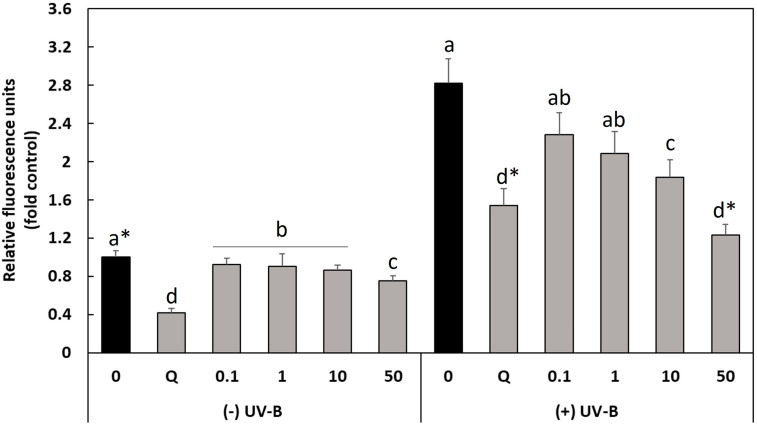
Relative fluorescence units (fold control) of HaCaT cells due to ROS produced in the absence and presence of UVB at 50 mJ cm^−2^ subjected or not to 24 h pretreatment with different concentrations of broccoli byproducts extract (BBE) (0, 0.1, 1, 10 and 50 μg mL^−1^). Q: Quercetin (positive control; 30 μg mL^−1^). Data are given as the average of two experiments with six replicates each ± SD. Different letters denote significant differences according to the Tukey test (*p* < 0.05). * *p* > 0.05. Q: Quercetin (30 µg mL^−1^).

Most of the skin damage that occurs from prolonged UVB radiation is associated with ROS and ROS-related inflammation, so the potential cytoprotective effect of the BBE on oxidative stress induced by UV-light was evaluated to elucidate the underlying protective mechanisms in HaCaT cells. Firstly, to confirm the non-toxicity of the BBE and the toxicity of UVB light, we conducted a cell viability assay of the cytotoxic effect of BBE and UVB light, separately or in combination (Figure 1). As can observed, only at low concentrations of BBE (0.1 and 1 μg mL^−1^), cell viability was significantly reduced compared to the untreated cells (0 μg mL^−1^) but in any case, cell viability was lower than 85%. As expected, exposure to UVB (50 mJ cm^−2^) significantly reduced cell viability (around 40%) and no statistical differences with respect to control cells was observed after the treatment with BBE at any concentrations. Based on these results, the intracellular ROS levels were evaluated using a fluorescent probe DHCF-DA. The fluorescence values obtained after exposure of the HaCaT cell monolayer to UVB light (50 mJ cm^−2^) with or without pretreatment with BBE are shown in Figure 2.

The results indicated that both the concentration of BBE and exposure to UVB radiation (50 mJ cm^−2^) individually or in combination were able to affect significantly intracellular ROS levels (*p*-value = 0.000). Thus, as shown in Figure 2, exposure to UVB light (50 mJ cm^−2^) significantly increased ROS generation, being about 2.8 times higher than for non-irradiated cells. However, in cells irradiated with UVB light, pretreatment with the BBE significantly reduced intracellular generation of ROS at all tested concentrations (from 0.1 to 50 μg mL^−1^), being the greatest reduction in intracellular ROS levels observed after pretreatment of HaCaT cells with 50 μg mL^−1^ BBE and exposure to UVB light, reaching the basal levels of the control cells not exposed to any type of UVR or to the extracts. Under these conditions, the basal ROS levels of control cells were reached, that is, those cells not exposed to UV radiation or BBE, indicating that BBE acts as a free radical scavenger, demonstrating the protective effect of BBE against UVB-induced oxidative damage in HaCaT cells.

### 3.2. Effect of BBE on the Expression of Inflammation-Related Genes in HaCaT Keratinocytes Exposed to UVB Light

**Figure 3:**
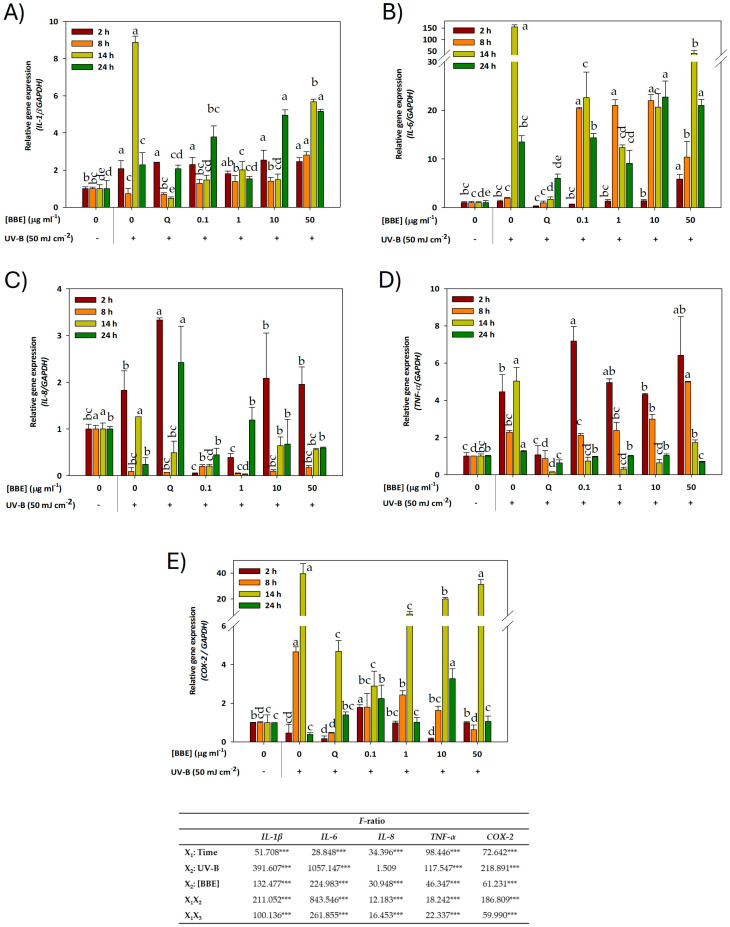
Relative expression (fold above control) of genes encoding proinflammatory cytokines IL-1β (**A**), IL-6 (**B**), IL-8 (**C**), TNF-α (**D**) and the cyclooxygenase COX-2 (**E**) in HaCaT cells pretreated with different concentrations of broccoli byproducts extract (0.1, 1, 10 and 50 µg extract mL^−1^) after 2, 8, 14 and 24 h of exposure to 50 mJ cm^−2^ of UVB light. Q: Quercetin (positive control; 30 μg mL^−1^). Values show mean ± SD of three independent replicates. UVB control (-) with reference value = 1 was used to normalize the relative expression levels of each gene. Transcript levels were calculated using GAPDH as a housekeeping gene. Two-way ANOVA F-values significantly at 99.9% (***). Different letters denote significant differences (*p* < 0.05) at each time.

To assess whether pretreatment with BBE was able to reduce oxidative stress caused by exposure to UVB light in HaCaT cells, the transcription profiles of some genes coding for different pro-inflammatory cytokines (IL-1β, IL-6, IL-8 and TNF-α) and the cyclooxygenase COX-2 were determined by qRT-PCR. The effect of different concentrations of BBE on the expression of UVB light-induced inflammatory mediators in HaCaT cells is shown in Figure 3 and Figure S3 (Supplementary Materials). The ANOVA analysis revealed that both time and the concentration of BBE as well as UVB radiation significantly affected gene expression of the *IL-1β* (Figure 3A), *IL-6* (Figure 3B), *TNF-α* (Figure 3D) and *COX-2* (Figure 3E). However, UVB irradiation did not affect the expression of the *IL-8* gene (Figure 3C) (*p*-value = 0.224), although the expression of this gene was statistically affected by both time and the extract concentration used. In addition, the interaction of different factors (time × concentration of extracts and time × UVB radiation) also showed significant effects on the expression profile of all genes studied (*p*-value = 0.000). In addition, the effect of the BBE without UVB radiation on the expression of studied genes, in most cases, did not significantly affect their expression (Supplementary Figure S3). Regarding the analysis of the *IL-1β* gene expression profile (Figure 3A), a significant increase in the accumulation of transcripts was clearly observed after exposure to UVB light, both in untreated cells and in those treated with different concentrations of BBE. Thus, after exposure of the keratinocytes to UVB light, the maximum level of expression of the *IL-1β* gene reached 14 h from irradiation, at which time the accumulation of transcripts turned out to be nine times higher than the control without irradiation at that time. Considering the maximum expression of the *IL-1β* gene after exposure to UVB light (14 h), it can be observed that pretreatment with BBE concentrations ≤ 50 µg mL^−1^ significantly reduced the expression of this gene being six times lower than the control treatment (exposed only to UV light) and slightly higher than the effect observed after pretreatment with quercetin at that time. Furthermore, the highest concentration of BBE used (50 μg mL^−1^) produced a significant increase in the expression level of the *IL-1β* gene after 14 h exposure to UV light, but this increase was even smaller than that observed after UV light exposure in the absence of BBE. Similarly, the gene expression profile of the IL-6 proinflammatory cytokine was analyzed (Figure 3B). As occurred with the expression of the *IL-1β* gene, treatment with UVB light considerably increased the level of expression after 14 h from irradiation, reaching at that point, a transcript accumulation about 150 times higher than non-irradiated cells. In addition, at this time, the expression of the *IL-6* gene decreased significantly after pretreatment with different concentrations of BBE, this decrease being more pronounced after pretreatment with 1 μg mL^−1^ of BBE, whose accumulation of transcripts was 12.5 times lower than keratinocytes exposed to UV light alone, although in no case was the level of protection induced by quercetin reached.

On the other hand, the expression level of the gene encoding the cytokine IL-8 was also studied (Figure 3C). As observed in the figure, the expression of the *IL-8* gene showed a variable expression profile with the different treatments. Thus, as previously mentioned, this gene was the only one that was not significantly affected by exposure to UVB light, as demonstrated by the analysis of variance (*p* value = 0.224). However, as can be seen in Figure 3C, exposure to UV light in the absence of BBE gave rise to two maximum expression peaks, at 2 and 14 h after irradiation. In this sense, after 2 h from irradiation, the pretreatment with low concentrations of BBE (0.1 and 1 µg mL^−1^) showed a significant decrease in the level of expression of the *IL-8* gene, almost completely inhibiting its expression, while after pretreatment with 10 and 50 µg mL^−1^ of BBE, no significant differences were observed compared to control cells (only exposed to UV light). Similarly, after 14 h from irradiation, the pretreatment of keratinocytes with the BBE, at any concentration, resulted in a significant reduction in gene expression, this decrease being more pronounced after pretreatment with 1 µg mL^−1^ of BBE, being the accumulation of transcripts 12 and 5 times lower than that obtained in cells exposed only to UV light and pretreated with quercetin, respectively.

We also evaluated the ability of the BBE to modulate *TNF-α* gene expression in HaCaT cells after UVB irradiation (Figure 3D). As in the previous cases, *TNF-α* gene expression also increased after irradiation of keratinocytes with UVB light, with two maximum expression peaks observed at 2 and 14 h after irradiation. Thus, after the first 2 h, an increase in gene expression of up to 4.45 times was observed in irradiated cells compared to those not exposed to UV light. This increase was maintained in keratinocytes pretreated with BBE and irradiated with UVB light, but this did not occur in cells treated with quercetin, which presented a greater effectiveness in reversing the induction of TNF-*α* gene expression after exposure to UV light, reaching expression values like untreated cells. However, 14 h after exposure to UV light, the five-fold increase in *TNF-α* mRNA expression induced by UVB light was strongly down-regulated by the BBE at any concentration, and almost completely restored after pretreatment with the BBE, maintaining this effect until 24 h. Therefore, pretreatment with BBE, even at low concentration, is sufficient to reverse the already established inflammatory state.

Finally, the expression profile of the gene encoding the cyclooxygenase COX-2 was studied (Figure 3E). As shown in this Figure, the exposure of keratinocytes to UVB light significantly increased the expression of this enzyme, with the highest accumulation of transcripts occurring 8 and 14 h after irradiation. At these times, COX-2 gene expression levels were 4.6 and 39 times higher than in cells not exposed to UVB light, clearly indicating an inflammatory response after UVB irradiation in HaCaT cells. The increase in *COX-2* gene expression observed after 8 h of exposure to UV light was significantly reversed after pretreatment with BBE at all tested concentrations, with the greatest effect observed when it was applied at a concentration of 50 µg mL^−1^, also showing a similar effect to that obtained after pretreatment with quercetin, whose expression levels were reduced by around nine times compared to control cells (exposed to UVB light in the absence of pretreatment). On the contrary, after 14 h from irradiation, the inflammatory effect induced by UVB light was only partially reversed by pretreatment with BBE since an increase in the level of *COX-2* gene expression was observed as the concentration of BBE increased, being in this case the concentration of 0.1 µg mL^−1^ the one that showed greater efficacy against the stress caused by UV light, reducing gene expression around 1.64 and 14 times compared to cells pretreated with quercetin and control cells, respectively.

### 3.3. Use of BBE to Repair Skin Wounds

**Figure 4:**
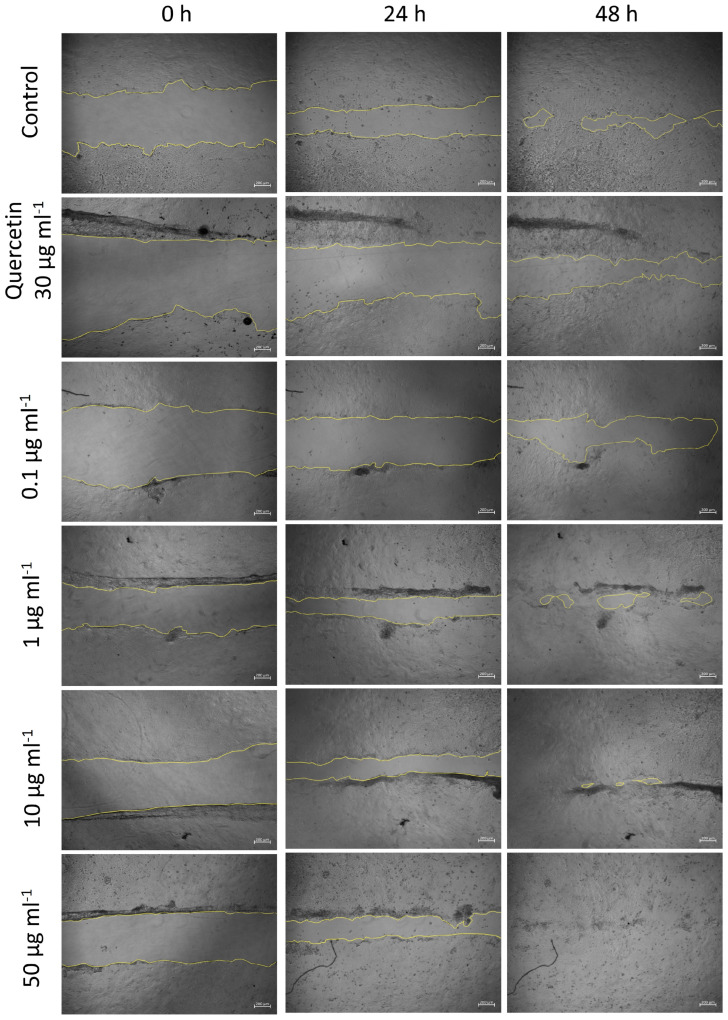
Phase contrast microscopy images of wound closure assays in HaCaT cells at different times (0, 24 and 48 h) after 24 h of pretreatment with different concentration of broccoli byproducts extract (0.1, 1, 10 and 50 µg mL^−1^). Quercetin (positive control; 30 µg mL^−1^). Yellow highlighted lines define wound edges. Scale bar: 200 µm.

**Figure 5:**
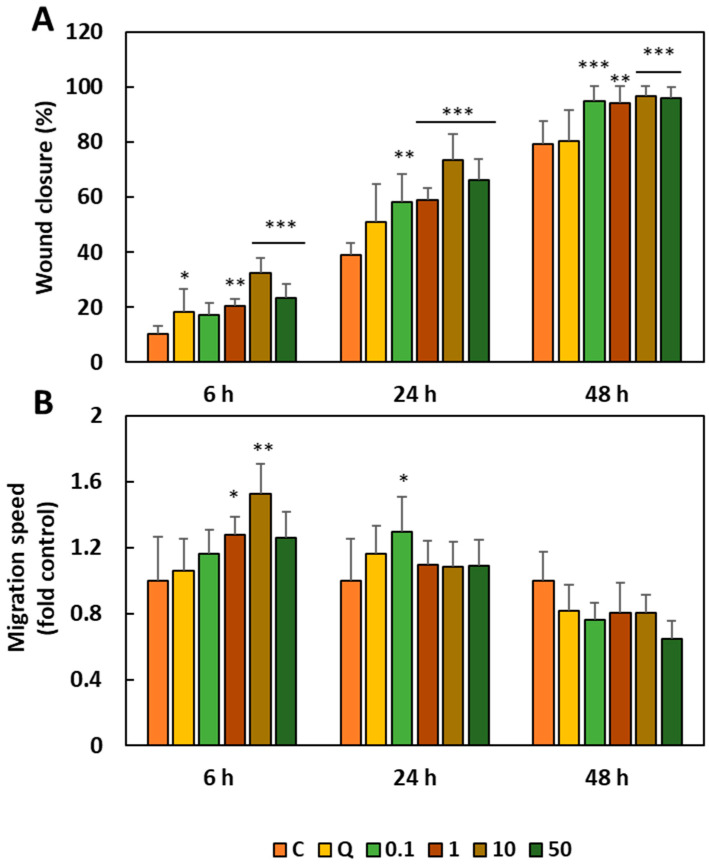
Percentage of wound healing (**A**) and migration speed (fold control) (**B**) of HaCaT cells after 6, 24 and 48 h with a 24 h pretreatment with broccoli byproducts extract (0.1, 1, 10 and 50 µg mL^−1^) before wounding. C, control; Q, quercetin (positive control; 30 μg mL^−1^). Results are expressed as the meaning of two assays with four replicates ± SD. * Significant coefficients (* *p*-value < 0.05; ** *p*-value < 0.01 and *** *p*-value < 0.001).

To evaluate the wound repair effect of BBE, a cell migration test was carried out in HaCaT cells pretreated with different concentrations of BBE (0.1, 1, 10 and 50 µg mL^−1^) for 24 h. After this time, each cell monolayer was wounded in a straight line using a sterile sharp agent and kept in incubation for 48 h, taking photographs at 6, 24 and 48 h to determine the wound closure length (mm) (Figure 4). Additionally, quercetin was used as a positive control at a concentration of 30 µg mL^−1^. The results obtained from the analysis of wound healing in keratinocytes pretreated with different concentrations of BBE (Figure 3) showed that, as the extract concentration increased, the wound healing process became more evident, with complete wound healing observed after 48 h in cells pretreated with 50 µg mL^−1^ BBE. In addition, the healing process occurred more quickly than in keratinocytes treated with quercetin (positive control). These data were corroborated after determining the healing percentage (Figure 5A) in which it was observed that all concentrations of BBE used resulted in wound closure areas that were statistically significantly higher than in untreated cells, with this wound healing effect again being more evident at 48 h after wound creation. After this time, the control cells showed a healing percentage of around 79%, very similar to that presented by the cells exposed to quercetin, which closed the wound by 80%. However, all the concentrations of BBE used presented a healing closure percentage close to 100%, being the percentages of 95, 94, 96 and 96%, for the concentrations of 0.1, 1, 10 and 50 µg mL^−1^, respectively. As expected, the migration speed of the cells (Figure 5B) was higher after 6 h from the creation of the wound because at that time the cells were in the active growth phase, observing at that time, a higher healing speed of the cells treated with concentrations < 1 µg mL^−1^ with respect to the control cells and those treated with quercetin.

## 4. Discussion

UVB rays absorbed by epidermal cells cause damage to DNA, increase oxidative stress, ROS and lead to premature aging of the skin [21]. ROS are considered one of the most studied and described indicators of oxidative stress in human cells [22]. Thus, ROS generation induced by UV radiation plays a very important role in skin aging, inflammation and photo carcinogenesis as an excessive production of ROS can break the pro-oxidant/antioxidant cellular balance and cause oxidative stress in skin keratinocytes and fibroblasts [23,24]. The harmful effects of ROS on the skin are generally neutralized by the body’s antioxidant defense mechanism, which consists in the activation of antioxidant enzymes and free radical-scavenger molecules. Numerous studies have shown that plant extracts with antioxidant properties can mitigate ROS generation by reducing UV-induced photo aging and skin photo carcinogenesis [25]. Here, we have demonstrated the ability of BBE to reduce UVB-induced oxidative damage in the HaCaT keratinocytes. Similar results were obtained by Jaisin et al. [26], who observed a significant increase in ROS levels, up to 1.6 times more than the control, in HaCaT cells exposed to 40 mJ cm^−2^ UVB radiation, being able to reverse the effect of UV light up to control levels when cells were treated with piperine. Other antioxidant compounds such as afzelin or fisetin also showed the same reducing effect on ROS generation, in a dose-dependent manner, in cells exposed to UVB light (20 mJ cm^−2^) and hydrogen peroxide, respectively [27,28].

The acute skin response to UV exposure is inflammation, such as erythema and edema and mitochondrial and DNA damage caused by ROS [29]. ROS are a byproduct of oxygen metabolism and are involved in many physiological functions, such as cellular signaling, activation of pathogen defense mechanisms and cell proliferation. However, skin exposure to UV rays can produce higher amounts of ROS, which causes an imbalance between the production of these radicals and the antioxidant defense mechanisms, generating oxidative stress. This oxidative stress can increase the production of ROS by initiating both inflammation and activation of pro-inflammatory cytokines such as IL-2, IL-6 and TNF-α, which involve multiple pathways, including the activated nuclear factor kappa B (NF-kB), among others [21,30]. All these compounds generated during inflammation act as oxidants and promote the appearance of ROS, so they increase oxidative stress, becoming a kind of vicious circle. All together they play an important role in the development of skin cancer since it can transform a benign solar keratosis into a squamous cell carcinoma that would increase oxidative damage to DNA [3,31]. The results obtained here have shown that UVB radiation (50 mJ cm^−2^) is capable of significantly affecting gene regulation encoding for the proinflammatory cytokines studied, IL-1β, IL-6, TNF-α and the cyclooxygenase COX-2. This contrasts with other studies in which irradiation of HaCaT cells with UVB light (with doses ranging from 5 to 100 mJ cm^−2^) produced a positive effect on gene regulation of these molecules involved in inflammatory processes [6,26,32,33]. This may be because the expression and secretion profile of cytokines is dependent on both the dose of UV radiation applied and other variable conditions such as the type of cells, number of subcultures, culture time, etc. [5].

On the other hand, there are numerous studies describing the potential activity of both pure compounds and complex plant extracts to attenuate the expression of these inflammatory markers in skin cells exposed to UVB radiation. Some examples are piperine (an alkaloid with antioxidant properties found in pepper), which has shown efficacy in decreasing the expression of IL-6 and IL-8, as well as inhibiting the synthesis of COX-2/prostaglandins (PGE2) [22]; fisetin, which is capable of inhibiting the production of nitric oxide (NO), PGE2, IL-1β, IL-6, as well as the expression of iNOS and COX-2 and the activation of NF-κB in HaCaT cells, in this case, treated with TNF-α [28]; flavonoids such as afzelin also show the ability to inhibit lipid peroxidation and cyclooxygenases (COX-1 and COX-2) induced by UVB light, as well as being able to reduce the expression of IL-6 and TNF-α in the same cells [27]. Other compounds such as rosmarinic acid have also shown a protective effect, significantly reducing the expression of IL-6, IL-8, MCP-1 and TNF-α [32]; curcumin which is also capable of down-regulating the expression of proinflammatory cytokines such as IL-1β, IL-6 and TNF-α [34] or nicotinamide (the amide form of vitamin B3, present in broccoli) which was able to reverse the increase in the expression of IL-6 and TNF-α in keratinocytes exposed to UVB radiation [6]. Likewise, the use of plant extracts enriched in compounds with antioxidant activity has also been investigated for use as therapeutic agents because they are capable of alleviating inflammatory processes of the skin. Thus, studies carried out by Engel et al. [35] demonstrated that topical treatment with extracts of *Usnea barbata* in inflammatory skin conditions caused by UVB radiation could be beneficial, since its use on HaCaT keratinocytes caused a decrease in PGE2, as well as the COX-2 protein. Likewise, the use of ethanolic extracts of *Sanguisorba officinalis* L. in keratinocytes reduced the expression of IL-8 at both the protein and mRNA levels [36], or pomegranate extracts enriched in ellagitannins that were able to inhibit the production of free radicals in the skin and decrease the expression of COX-2, protecting it from DNA damage and thus reducing the risk of skin cancer [37]. *Laminaria japonica* extracts were also able to decrease the expression of COX-2, metalloproteinase-9 (MMP-9) and PGE2, as well as cytokine IL-8 and TNF-α, induced by UVB radiation [33]. Fig leaf extracts (rich in polyphenols and antioxidant compounds) were also able to reduce the expression levels of inflammation-related genes such as TNF-α [38]. There are even clinical studies that have proven the beneficial effect of quantified compounds in our extracts, such as dl-α-tocopherol, which have photoprotective effects against the damage caused by UVB radiation [39,40] or the topical application of an essential oil from *Curcuma longa* leaves that was able to inhibit skin edema, in addition to reducing the production of TNF-α, IL-6 and IL-1β, in mouse ears [41], as occurred with an extract rich in flavonoids from *Sophora flavescen* [42]. In accordance with all these results, we can indicate that the topical use of extracts rich in bioactive compounds with antioxidant capacity may be beneficial for the treatment of inflammatory skin disorders such as those produced by exposure to UVB light. Taken together, these studies indicate that plant extracts rich in bioactive and antioxidant compounds, such as BBE, are able to reduce oxidative stress and inflammation in the skin produced by UVB light. Thus, the results obtained suggest that the BBE can prevent and/or limit the inflammatory cascade induced by exposure to UVB radiation, through a reduction of pro-inflammatory mediators, so we could consider this extract as a promising natural cosmetic ingredient for use as skin protectors against the harmful effects produced by UVB radiation.

However, despite the promising outlook for plant byproducts in cosmetic use, there are some limitations that need to be addressed. An important aspect to consider when valuing the bioactivity of plant extracts is the presence of pesticides when conventional cultivation methods have been used. In this sense, conventional methods use chemical pesticides and other products not used in organic crops. Although no specific studies have been carried out on pesticide residues in broccoli crops, in other plant species, it has been found that 30% of the parts intended for consumption contained pesticides when conventional cultivation methods were used. Therefore, in addition to the characterization of phytocompounds, further work on the presence of possible pesticides in the byproducts should be carried out to ensure health promotion.

As mentioned above, keratinocytes are cells that are generated at the base of the epidermis and ascend towards the most superficial layer and play an essential role in the protection of this organ, as they form a physical barrier against infection and hydroelectrolytic loss. In addition to forming a physical barrier, keratinocytes actively respond to pathogenic microorganisms and injuries, producing antimicrobial peptides and various cytokines, which promote and regulate immune responses. Therefore, in processes related to wound healing, keratinocytes are the main cells responsible for the epithelialization phase of wound healing as they migrate to the affected area where they proliferate to contribute to the wound-healing process [43]. Historically, in traditional medicine, numerous plants have been used as promoters of wound healing [41] since, in general terms, plant extracts usually show fewer side effects than synthetic compounds, in addition to presenting important biological activities. Similarly, we have demonstrated that the extracts enriched in bioactive compounds obtained by SFE from broccoli byproducts could be an excellent potential therapeutic agent for skin wound healing. In this sense, numerous plant extracts rich in bioactive compounds have been studied to verify their efficacy in wound repair. Such is the case of extracts from the flower of *Smilax china*, which shares with the broccoli extracts obtained by SFE some compounds such as hexadecanoic acid, (9Z,12Z,15Z)-octadeca-9,12,15-trienoic acid or β-sitosterol. The application of these floral extracts increased the migration rate of HaCaT cells in a dose-dependent manner, in addition to significantly increasing the synthesis of type I and IV collagen, both involved in skin repair and membrane formation, respectively [43]. Other extracts obtained from the stem of *Alternanthera sessilis*, also rich in hexadecanoic acid and phytol, showed the same effect. In this case, the treatment of damaged keratinocytes with these extracts at a concentration of 50 µg mL^−1^ showed an increase of up to 209% in the cell migration rate compared to control cells [44]. In similar trials using extracts obtained from broccoli, Nicolas-Espinosa et al. [45] demonstrated an increase in the percentage of wound closure in the presence of these extracts. Furthermore, in this research, the expression profile of genes involved in cell proliferation, apoptosis and inflammatory processes was also studied after the application of extracts. The results indicated that exposure of keratinocytes to broccoli stem extracts resulted in an overexpression of inflammatory response genes and cell growth-promoting molecules related to cell proliferation and wound repair. However, even though plant extracts have been proven in several vivo and clinical trial studies for their potential use as remedies to heal wounds [46], there are some limitations to their use regarding safety, stability, solubility and limited activity on the wound site. Therefore, the design of formulations that will allow for the development of a cost-effective, efficient, stable and sustainable delivery system for wound treatment will open new research ways in wound care management.

As has been demonstrated, in this work, the use of BBE as a source of bioactive compounds for cosmetic purposes represents the most significant interest in valorization. Therefore, since the demand for the application of bioactive compounds from plant byproducts in skin care products is growing, it is mandatory to prove their safety and efficacy through well-conducted in vivo and clinical trial studies as well as to understand the molecular mechanisms underlying the observed effects.

## 5. Conclusions

The present study have demonstrated that BBE have potential utility as additives in cosmetic products since it can effectively reduce the intracellular content of ROS induced by UVB radiation offering protection against oxidative stress damage, as well as effectively reversing the increase in proinflammatory cytokines induced by UVB radiation (IL-1β, IL-6, IL-8, TNF-α) and the cyclooxygenase COX-2 in HaCaT cells. Likewise, BBE could be an excellent potential regenerative agent for skin wound repair, as demonstrated by the results of the wound healing study in keratinocytes pretreated with BBE which accelerate the migration of skin keratinocytes. In summary, we can consider BBE as a promising natural cosmetic ingredient for use as a skin protector against the harmful effects produced by UVB radiation as well as promising wound heal, thus contributing to the valorisation of this byproduct as a valuable ingredient in cosmetic formulations.

## Figures and Tables

**Table 1:** List of gene-specific primers designed using PrimerQuest™ Tool software (https://www.idtdna.com/Primerquest/Home/Index, accessed on 15 May 2022) for real Time RT-PCR.

Target Gene	GenBank^®^ Accession Number	Primer Pairs (5′-Forward-3′/5′-Reverse-3′)	Fragment Size (bp)
*GAPDH*	NM_002046	TGAGCATCTACGGTTTGCTG/TGCTTGTCTGGAACAACTGC	202
*IL-1β*	NM_000576	GCAACCGCTTCCCTATTTAT/TGCTTGTCTGGAACAACTGC	90
*IL-6*	NM_000600	ACCTTCCAAAGATGGCTGAA/TGGCTTGTTCCTCACTACTC	146
*IL-8*	BC013615	GTTAAATCTGGCAACCCTAG/GGTAAGATGGTGGCTAATAC	111
*TNF-α*	NM_000594	CAGGGACCTCTCTCTAATCA/TGCTACAACATGGGCTACAG	89
*COX-2*	M90100	TTGACAGTCCACCAACTTAC/GAGGAAGGGCTCTAGTATAA	88

## Data Availability

The original contributions presented in this study are included in the article and Supplementary Materials. Further inquiries can be directed to the corresponding author.

## Supplementary Materials

The following are available online at https://www.mdpi.com/article/10.3390/antiox13121479/s1, Figure S1. HPLC-DAD profile of the optimized supercritical fluid (SFE) extracts derived from broccoli byproducts, acquired at 454 nm. Figure S2. Total ion chromatogram (TIC) of the optimized supercritical fluid (SFE) extracts derived from broccoli byproducts. (b) Identified compounds in the extracts obtained from broccoli byproducts. * denotes the quantified compounds. Figure S3. Heatmap of the expression of genes encoding proinflammatory cytokines (IL-1b, IL-6, IL-8 and TNF-a) and cyclooxygenase COX-2 in HaCaT cells pretreated with different concentrations of BBE (0.1, 1, 10 and 50 µg extract mL^−1^) exposed (UVB+) or not (UVB-) to UV light after 2, 8, 14 and 24 h, after normalization against the control (C). Asterisks denote non-significant differences against the control without UV light treatment at each time point (*, *p* > 0.05). FC: fold change.
