# Supplementary material for: Broccoli Byproduct Extracts Attenuate the Expression of UVB-Induced Proinflammatory Cytokines in HaCaT Keratinocytes
Source: Antioxidants (Basel). 2024 Dec 2;13(12):1479. doi: 10.3390/antiox13121479 (PMC11673147; doi:10.3390/antiox13121479)
Supplement: Supplementary file 1 [file antioxidants-13-01479-s001.zip › Figure S3.pdf]

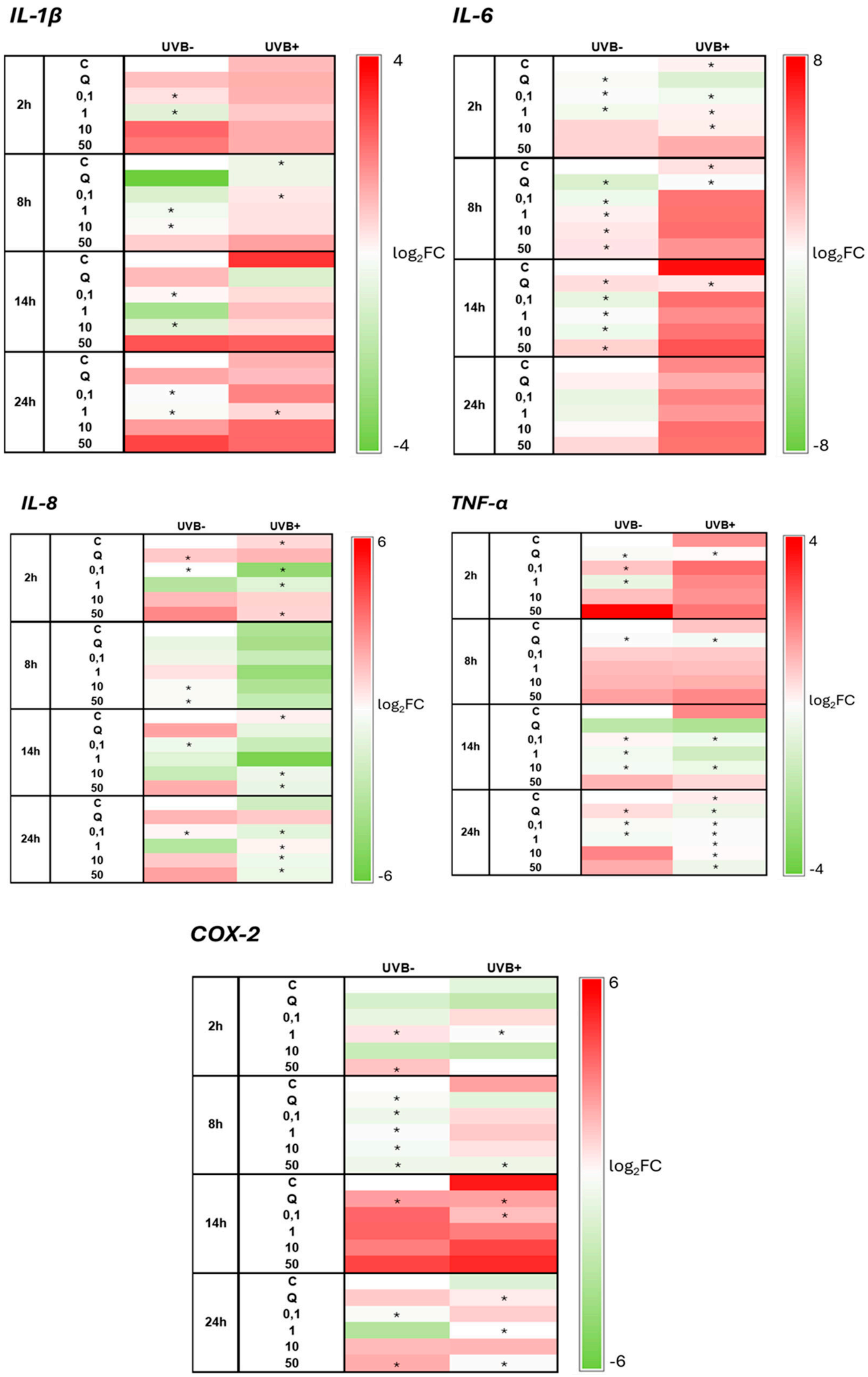

Figure S3. Heat map of the expression of genes encoding proinflammatory cytokines (IL-1b, IL-6, IL-8 and TNF-a) and cyclooxygenase COX-2 in HaCaT cells pretreated with different concentrations of BBE (0.1, 1, 10, and 50 µg extract ml^{-1}) exposed (UVB+) or not (UVB-) to UV light after 2, 8, 14 and 24 h, after normalization against the control (C). Asterisks denote non-significant differences against the control without UV light treatment at each time point (*, p>0.05). FC: fold change.
