# Supplementary figures and images for: Broccoli Byproduct Extracts Attenuate the Expression of UVB-Induced Proinflammatory Cytokines in HaCaT Keratinocytes
Source: Antioxidants (Basel). 2024 Dec 2;13(12):1479. doi: 10.3390/antiox13121479 (PMC11673147; doi:10.3390/antiox13121479)

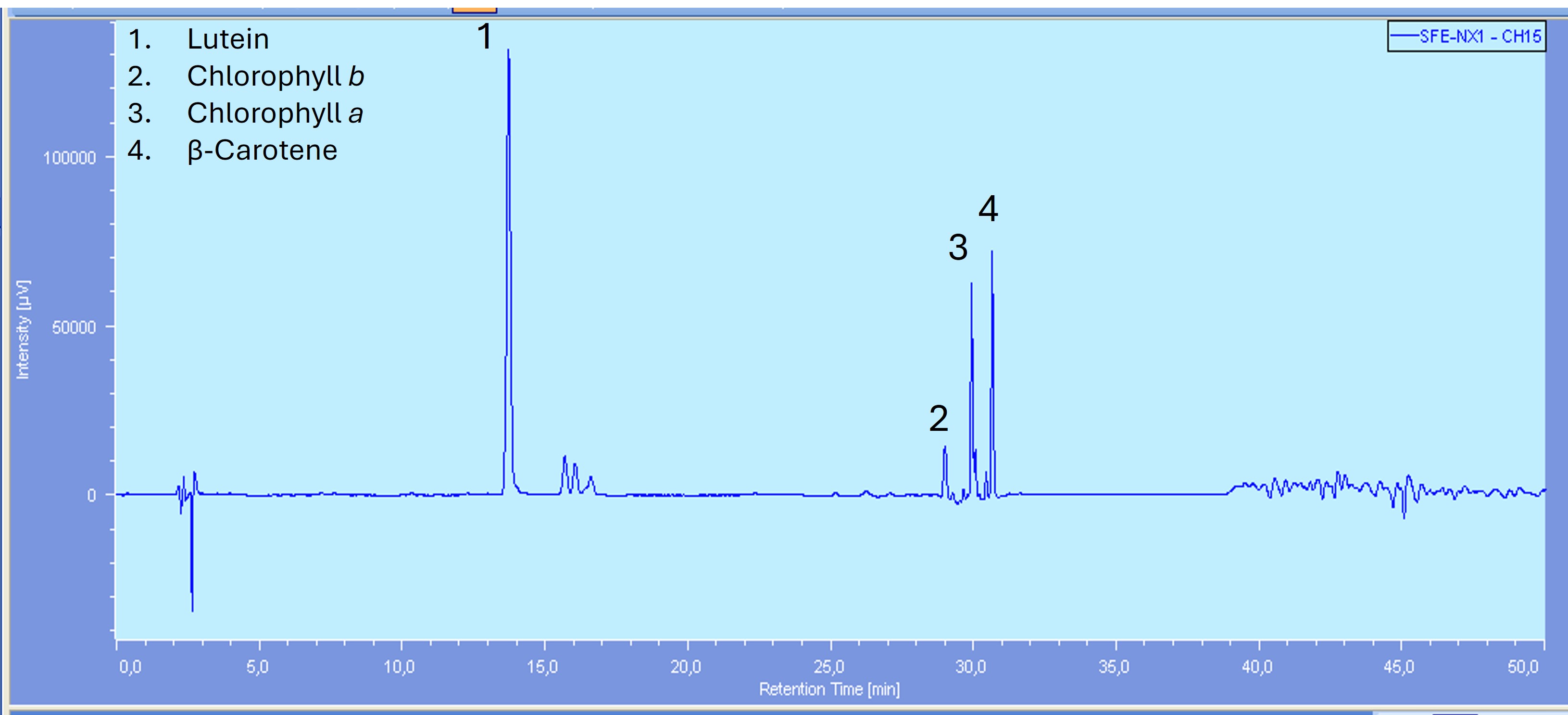

Supplement: Supplementary file 1 [file antioxidants-13-01479-s001.zip › Supplementary S1.jpg]

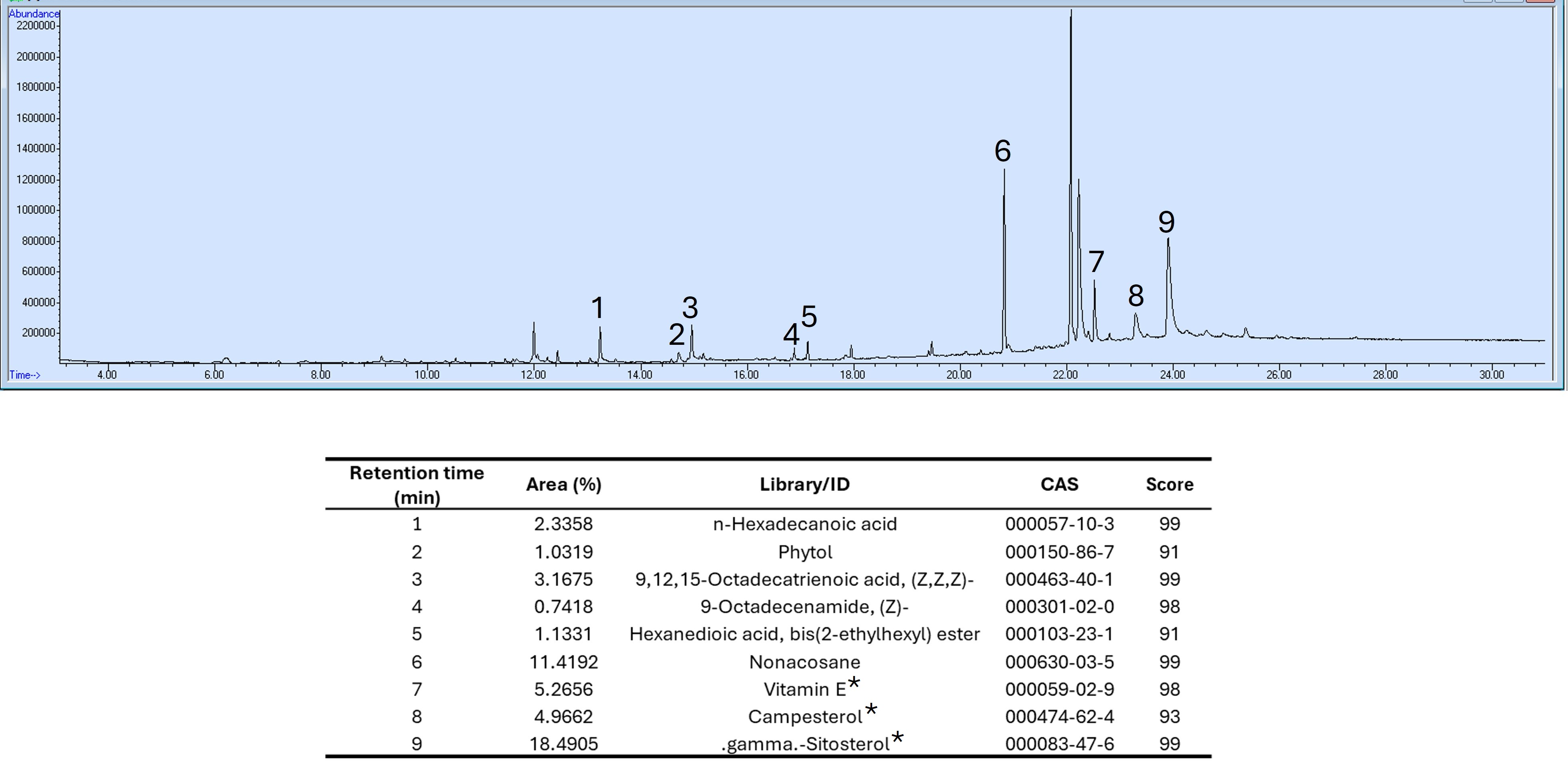

Supplement: Supplementary file 1 [file antioxidants-13-01479-s001.zip › Supplementary S2.jpg]
